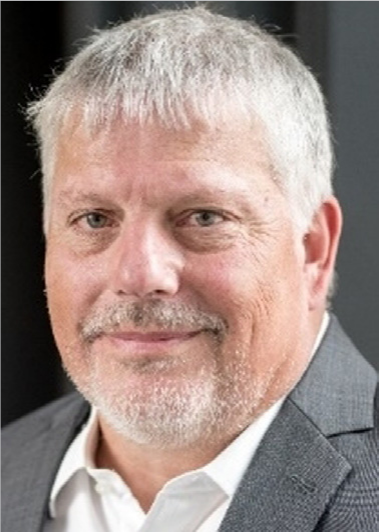# Electrocatalysis as the Nexus for Sustainable Renewable Energy: The Gordian Knot of Activity, Stability, and Selectivity

**DOI:** 10.1002/anie.202007672

**Published:** 2020-08-18

**Authors:** Justus Masa, Corina Andronescu, Wolfgang Schuhmann

**Affiliations:** ^1^ Max Planck Institute for Chemical Energy Conversion Stiftstrasse 34–36 45470 Mülheim an der Ruhr Germany; ^2^ Faculty of Chemistry Technical Chemistry III University of Duisburg-Essen Carl-Benz-Str. 201, ZBT 241 47057 Duisburg Germany; ^3^ Analytical Chemistry—Center for Electrochemical Sciences (CES) Faculty of Chemistry and Biochemistry Ruhr University Bochum Universitätstr. 150 44780 Bochum Germany

**Keywords:** activities, electrocatalysis, nanoelectrochemistry, selectivities, stabilities

## Abstract

The use of renewable energy by means of electrochemical techniques by converting H_2_O, CO_2_ and N_2_ into chemical energy sources and raw materials, is the basis for securing a future sustainable “green” energy supply. Some weaknesses and inconsistencies in the practice of determining the electrocatalytic performance, which prevents a rational bottom‐up catalyst design, are discussed. Large discrepancies in material properties as well as in electrocatalytic activity and stability become obvious when materials are tested under the conditions of their intended use as opposed to the usual laboratory conditions. They advocate for uniform activity/stability correlations under application‐relevant conditions, and the need for a clear representation of electrocatalytic performance by contextualization in terms of functional investigation or progress towards application is emphasized.

## Graphical abstract

**Water, nitrogen, and carbon dioxide** are “magic” molecules whose efficient electrocatalytic conversion into suitable energy can deliver limitless renewable energy. Discussed in this Minireview are the key fundamental and experimental factors that aid in the understanding of the dynamic nature of active sites, reaction kinetics, and mechanisms, as well as selectivities and catalyst stabilities on a molecular level.

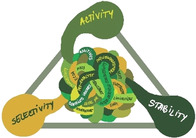

## 1 Introduction

At a simple electrochemical interface, a tiny volume of space at the intersection of an electrode and an electrolyte, proceed complex chemical phenomena, including adsorption and desorption processes, electron‐ and charge‐transfer reactions, solvation and desolvation, and electrostatic interactions, among others, that can be exploited to supply limitless energy. Engineering and tailoring reactions at electrode/electrolyte interfaces is the foundation of present‐day versatile energy storage that include batteries and electrochemical supercapacitors,^1^ fuel cells,^2^ as well as many important electrochemical syntheses. The reactions at this interface are sensitive to surface electronic and geometric properties of the electrode, electrolyte properties (including pH value, concentration, ionic strength, and the nature of anions and cations), and the electric field strength to mention a few.^3^, ^4^

An ideally designed electrochemical interface, which is the goal of electrocatalysis, would make it possible to exploit the excess energy harnessed from renewable sources to convert molecules at our everyday disposal, H_2_O, N_2_, and CO_2_, into limitless energy carriers for energy applications from light portable systems and electromobility to mini‐grid‐ and grid‐scale energy storage,^4^, ^5^, ^6^ as well as industrial chemicals.^5^, ^7^ For example, reversible interconversion of H_2_O into H_2_ in an electrolyzer, and the recombination of H_2_ and O_2_ in a fuel cell to reform H_2_O, releasing useful energy, offers the prospect of a virtually limitless supply of clean energy and is the essence of the hydrogen economy.^8^ The selective electrochemical conversion of CO_2_ into a variety of useful fuels and chemicals, including hydrocarbons (e.g. methane or ethylene), alcohols (e.g. methanol, ethanol, and propanol), and other compounds, such as formic acid, among others,^9^ would substantially decrease our net CO_2_ emissions, while electrochemical N_2_ reduction to form NH_3_, would provide a green carbon neutral gateway for ammonia production.^10^, ^11^

From the thermodynamic viewpoint, the electrochemical interconversion of H_2_O into H_2_ and O_2_, CO_2_ and H_2_O into CH(O) compounds, and N_2_ and H_2_O into NH_3_, are all feasible, however, the turnover rates are often disappointingly low. To achieve meaningful conversion rates, present electrolyzers have to be operated at far higher voltage inputs than predicted by thermodynamics, which renders the processes to be extremely energy intensive. Moreover, where multiple products are possible as in the case of electrochemical CO_2_ reduction, selective formation of preferred products remains a great challenge. There is also the challenge of material instability where catalysts by themselves, or as part of system components are unable to sustain satisfactory performance levels for a reasonable time. In a nutshell, the three main challenges that render present electrochemical conversion of N_2_, H_2_O, and CO_2_ valuable energy carriers and chemical feedstocks are insufficient activity, poor product selectivity, and unsatisfactory stability.

The focus of most research in electrocatalysis over the past two decades has been on discovering new electrocatalysts or improving the properties of already known electrocatalysts to achieve superior performance, with activity being invariably the dominant parameter of interest, while stability and selectivity are relegated to parameters of secondary importance. It is a daunting challenge to keep abreast with all the important developments in electrocatalysis today given the overly high rate of publications. Moreover, disparities in experimental methods, conditions, and procedures, as well as data reporting metrics make this task even more complicated. The quest for a couple of mV less overpotential resulted in a gold‐medal race often without considering the complex impact of a multitude of parameters which includes among others, for example, rescaling of the potential to the reversible hydrogen electrode scale assuming that the pH value of a 1 m KOH solution is 14, thereby neglecting activity coefficients, or the comparison of high surface area electrodes at the same overall current by normalization to the electrode's footprint. To this end, a few experimental guidelines and benchmarks for reporting activity and stability for the oxygen reduction reaction (ORR),^12^, ^13^ the oxygen evolution reaction (OER),^14^, ^15^ the hydrogen evolution reactions (HER),^16^ and for the nitrogen reduction reaction (NRR),^10^ were proposed to facilitate the comparison of results from different laboratories. However, scientific investigations for pure fundamental inquisition and application‐oriented research cannot have common benchmarks. For example, the current density of 10 mA cm^−2^ first proposed by Weber and Dignam in 1984 as the OER benchmark for direct conversion of solar‐to‐H_2_ in a photoelectrochemical cell (PEC),^17^ and later promoted by Gorlin et al.^18^ and McCrory et al.,^14^ although extremely valuable for cross‐laboratory comparisons, is evidently inconsequential for conventional water electrolysis where much higher currents, ≥400 mA cm^−2^ for alkaline electrolyzers and ≥2 A cm^−2^ for proton exchange membrane (PEM) electrolyzers, are demanded. For similar reasons, the widely used stability benchmark of 10 mA cm^−2^ for 2 hours at room temperature is not a relevant metric for practical devices envisaged to operate in highly concentrated electrolytes for several thousand hours. Our rationale for highlighting these stark contrasts is to call for a change in perspective and scientific approach that will narrow the disconnection between important breakthroughs in materials discovery and their prospective industrial application. Continuing to study and compare thousands upon thousands of catalysts at these unrepresentative benchmarks will not bring us closer to the goal.

In this Minireview, we use experimental evidence to underscore the fact that for application‐oriented research, stability should not always be considered as the parameter of secondary importance to be optimized after active materials have been identified, but rather to refocus stability as an inherent part of the primary design approach. For fundamental investigations that seek to discover the composition and properties of the most potently active sites for specific reactions, we highlight methods and techniques, mostly based on nanoelectrochemistry, for accurate determination of intrinsic kinetic parameters and simple experiments for quick assessment of (electro)chemical stability. Some strategies for tailoring reaction selectivity are discussed in the context of emerging catalyst design concepts, including high entropy alloys (HEA), nanozymes, and electrocatalysis in micro‐ and nanoconfined volumes.

## 2 Activity, Stability, and Selectivity

Judging from the large number of publications claiming high activity, stability, and selectivity of electrocatalysts for key energy conversion reactions, but little evidence of translating such discoveries into practical utility, points to missing links in current research approaches and technological relevance of commonly reported experimental data. Electrocatalysis encompasses the design of catalysts, electrode‐electrolyte interfaces, and the reaction conditions to afford the highest faradaic turnover at minimum overpotential.

### 2.1 Missing Links to Accelerated Technological Applications

We have identified five factors, excluding common experimental errors and disparities in data treatment, such as correction for ohmic drop and charging currents, that we think are the critical missing links between fundamental materials design and implementation, and which may help to cross the so called “valley of death”, a largely unexplored region between fundamental electrocatalysis and technological applications.

*1) Performance and material behavior at application‐relevant conditions differ from those at laboratory conditions*

At the fundamental level, too much effort is devoted to material synthesis, in‐depth physicochemical characterization of the synthesized materials, which are in most cases pre‐catalysts that convert into the active material at operating conditions, and electrochemical testing at laboratory conditions. Evaluation of electrocatalytic performance and material behavior at laboratory conditions is based on the assumption that a similar behavior can be expected at application‐relevant conditions without drastic discrepancies. As will be shown later in Section 2.3.2, although true for some reactions and materials, experimental evidence reveals that contrastingly stark differences in both material behavior and electrocatalytic performance are observed between room‐temperature laboratory tests in low concentration electrolytes and high temperature in high concentration electrolytes. Presently, electrocatalytic performance is not only evaluated at unrepresentative conditions, but too much importance is given to catalytic activity. This is even more true since the measured activity is a system response of a complex catalyst‐modified electrode and does not in most cases reflect the intrinsic catalytic activity of the material itself. A material with moderate activity at laboratory conditions may be inherently more stable at application‐relevant conditions and even exhibit a higher activity in the longer‐term than a material that exhibits higher activity at laboratory conditions but is inherently less stable at application‐relevant conditions, resulting in rapid activity decay and thus comparatively lower activity in the longer term. Future research must therefore evaluate activity and stability interpedently at application‐relevant conditions and establish activity–stability relationships to guide the design of improved catalysts.

*2) Unrepresentative benchmarks and experimental conditions*

For hydrogen fuel cell reactions, the HOR and ORR, the benchmarks for specific activity at laboratory scale based on normalization of the current at a defined electrode (over)potential with the electrochemically active surface area of the catalyst (ECSA), catalyst mass (mass activity), or volume of catalysts, for the case of nonprecious metal catalysts (volumetric activity), were validated to be satisfactorily reproducible at industrial conditions.^12^ In addition, there are clear experimental protocols and performance targets, for example, those periodically published by the United State department of energy (DOE), that catalysts must fulfil to be considered for technical applications.

On the contrary, there are no well‐defined laboratory benchmarks for the HER and OER that are translatable to industrial scale. The HER is extremely fast at negligible overpotential making it difficult to extract mass transport free kinetic currents. Consequently, activity benchmarks for water splitting must be tagged to the OER, which is at least three orders of magnitude slower than the HER. The drawbacks for using the commonly adopted (over)potential at a geometric current density of 10 mA cm^−2^ as the activity metric for the OER and HER include:

1. The benchmark was initially proposed for the OER at the photoanode in direct photoelectrochemical water splitting without the application of any bias,^17^ which is a certainly very low current density for conventional electrochemical water splitting.
2. Since 10 mA cm^−2^ is not a kinetic current, and thus not an intrinsic kinetic parameter, it is prone to being influenced by many extrinsic factors, including the properties and roughness factor of the electrode, charging currents, and mass‐transport conditions.
3. Because of factors (1) and (2), the benchmark is unrepresentative of technical application conditions where the electrolyte concentration, temperature, and mass transport are much higher.

As mass‐transport and electrochemical double layer (EDL) properties are expected to be considerably different at high current densities, high electrolyte concentration, and high temperature, the missing link are the performance benchmarks on the laboratory scale that reliably depict the performance at application‐relevant conditions.

*3) Unsuitable characterization methods*

Thin‐film rotating disk electrode (RDE) voltammetry, the predominant method of choice for evaluating electrocatalytic performance, is for many reasons ill‐suited to study the kinetics of gas evolving reactions (e.g. HER and OER). RDE is designed to increase mass transport so that kinetic phenomena, in this case faradaic reactions, can be studied accurately without mass‐transport hindrances. For the HOR and ORR (gas consuming reactions) with well‐defined steady‐state mass‐transport limiting currents (*i*_L_), the kinetic current at a defined potential (*i*_K_), free of mass‐transport interference, can be either calculated from the Koutecky–Levich equation [Eq. (1)], or determined from a plot of the reciprocal of the total current (*i*) against the reciprocal of the square‐root of the rotation speed.

(1) $$i_{K}=\;\frac{{i\;\times \;i}_{L}}{i_{L}-i}$$

The adoption of RDE voltammetry to determine the activity of gas‐evolving reactions is a matter of convenience with its purpose limited to improving gas bubble removal to prevent electrode blockage. Thus, for these reactions RDE cannot serve its conventional purpose of determining kinetic parameters since mass transport free kinetic currents, and hence the turnover frequency (TOF), specific activity, and reaction rate constant, among others, cannot be accurately determined.

*4) Insufficient knowledge of true active sites and their dynamic stability*

Advances in tools and techniques for ex situ and in situ material characterization, as well as operando spectroscopies have enabled insights, at the atomic and molecular scales, into transient and steady‐state behavior of materials under electrochemical reaction conditions. However, the contribution of extrinsic factors, particularly, the solvent molecules (water) and the electrolyte ions (cations and anions), to the covalent and noncovalent interactions in the electrochemical double layer, and the ultimate effect on performance and stability is only beginning to attract broader attention. Besides the well‐recognized covalent electrode‐adsorbate bonds, previously overlooked weaker interactions of interfacial ions and solvent molecules with active sites and reaction intermediates are now being recognized to play a non‐negligible role on the mechanisms and kinetics of electrocatalytic reactions, as well as the stability of the active sites.^3^, ^4^

*5) Challenges of current normalization*

When determining the specific activity of nanoparticle film ensembles, the actual surface area of the catalyst involved in the reaction, the electrochemical active surface area (ECSA), must be known. The three methods that are most commonly used for estimation of the ECSA of catalysts are based on pore volume (BET), double layer capacitance, and faradaic charge during adsorption or desorption of specific species.^19^ Unfortunately, none of these methods is applicable to all catalyst types and each has its own inherent disadvantages.

Electrocatalytic currents are more commonly normalized against the geometric area of the electrode. However, the surface area of catalysts can differ by up to two to three orders of magnitude depending on their morphological properties, degree of defects, and porosity, among others, which implies that huge disparities may exist between the ECSA of a catalytic film and its geometric area. Moreover, variations in catalyst loading and film quality and the use of 3D instead of 2D electrodes as supports are likely to further affect the roughness factor of a catalyst film significantly. Catalytic trends are therefore prone to misinterpretation if geometric area normalized currents are used to compare catalysts whose surface areas vary widely. Hence, as a minimal prerequisite we suggest showing the linear sweep voltammograms with three *y*‐axes, comparing current density normalized with respect to the footprint of the electrode, the measured current, and the current density measured against the double‐layer charging capacitance derived from a not‐faradaic region of the voltammograms at varying scan rates.

### 2.2 Electrocatalytic Activity

Searching for the ideal or most active catalyst that can achieve electrocatalytic conversion at thermodynamic equilibrium potential, or with minimum reaction overpotential, is in the focus. However, given the complex and dynamic nature of the electrochemical interface, identifying the most potently active catalyst is only part of the problem. When considering the intrinsic activity of electrocatalysts, the most important figure of merit is the turnover frequency (TOF), which is the number of moles of a specific product generated per second per active site, that is: $TOF=i_{\eta }/znF$ , where *i*_*η*_ is the faradaic current at a specific overpotential (*η*), *z* is the number of electrons transferred during the conversion, and *n* is the number of moles of active sites. For some reactions, for example, gas‐consuming reactions, it may be more suitable to express the TOF of the catalyst with respect to the rate of conversion of a specific reactant. The other fundamental kinetic parameter in electrocatalysis is the exchange current density (*i*_o_), however, in practical terms, *i*_o_ cannot be reliably determined for all electrochemical reactions except the HER and hydrogen oxidation reaction (HOR).^20^ Since accurate determination of TOF requires explicit knowledge not only of the composition of the active sites but also the actual number of active sites involved in the reaction, a comparison of electrocatalysts in terms of their TOF for a given reaction makes it possible to identify the fundamental units from which the most intrinsically active materials can be derived in a bottom‐up design approach.

A readily accessible kinetic parameter for electrocatalytic reactions is the specific activity, which is the kinetic current (*i*_K_) recorded at a defined potential normalized with respect to the surface area of the electrode, or mass activity if normalized against the catalyst mass. As discussed in section 2.1, the determination of *i*_K_ free of mass transport and charging currents can be achieved quite reliably by RDE for gas‐consuming electrodes but is challenging for gas evolving electrodes.

#### 2.2.1 What would be an Ideal Electrocatalyst?

For a given half‐cell reaction, the ideal electrocatalyst would be one characterized by a high standard rate constant (*k* ^o^), high TOF, or a high exchange current density (*i*_o_) at the equilibrium potential. The HER and HOR on Pt represent a typical case of close to an ideal electrocatalyst. For both of these reactions, the current increases exponentially in accordance with the Butler–Volmer equation [Eq. (2)] if the electrode potential is either slightly decreased or increased from 0.0 V (versus RHE), the equilibrium potential of the hydrogen electrode.

(2) $$i_{k}=i_{o}\left(e^{\frac{\alpha_{c}nF}{RT}\eta }-e^{\frac{-\alpha_{a}nF}{RT}\eta }\right)$$

Here *i*_k_ is the kinetic current, *i*_o_ is the exchange current density, *α*_c_ and *α*_a_ are anodic and cathodic transfer coefficients of the cathodic and anodic reactions, respectively, *F* is the Faraday constant, *n* is the number of electrons involved in the reaction, *η* is the overpotential, *R* is the gas constant and *T* is the temperature. The near‐ideal reversibility of the 2H^+^/H_2_ redox exchange on Pt represents the ideal characteristics of a desirable catalyst for a given electrochemical reaction. However, the HER and HOR both involve the transfer of only two electrons and have only one intermediate (adsorbed hydrogen atoms). The kinetics of electrode reactions generally decrease with the number of intermediates and electron‐transfer steps involved. A requisite characteristic of all reactions that involve the formation of intermediate species is the breakage of bonds in the reactant(s) and subsequent formation of new bonds in the product(s). For these reactions, the reactants, intermediates, and products are at some instant physically bound on the electrode surface. The kinetics of such reactions as well as their mechanisms and selectivity depend on the properties of the electrode and the bonding strengths of the reactants, intermediates and products, in accordance with the Sabatier principle. This principle postulates that optimal catalysis occurs when the binding strength of the reactants, intermediates and products on the catalytic surface is neither too strong nor too weak.

Taking the model HER and HOR [Eq. (3)], the electrode potential (*E*) at any instant will be defined by the interfacial concentration of the 2 H^+^/H_2_ redox species in accordance with the Nernst equation [Eq. (4)], where *E* ^o^ is the standard equilibrium potential (0.0 V vs. RHE), *n* is the number of transferred electrons, and *p*_H2_ is the partial pressure of H_2_. On a Pt surface, the interconversion between the 2 H^+^ and H_2_ species in reaction (3) proceeds via weakly chemisorbed H atoms (H_ads_) as the intermediate species, with Δ*G*_H_≈0 eV,^21^ where Δ*G*_H_ is the free energy of adsorption of hydrogen.

(3) $$2\;H{}^{+}+2\;e{}^{-}\vec{\leftarrow }H{}_{2};\;E{}^{\circ }{}_{H{}^{+}/H{}_{2}}=0.0\;V{}_{\mathrm{RHE}}$$

(4) $$E=E^{o}+\;\frac{RT}{nF}ln\frac{{\left(a_{H+}\right)}^{2}}{p_{H_{2}}}$$

For the HER, Koper defined the minimum thermodynamic overpotential (*η*_T_) as the difference between the standard equilibrium potential of the thermodynamically least favorable reaction and the standard equilibrium potential of the overall reaction.^22^ If the reduction of protons to form H_ads_, that is H^+^+e^−^→H_ads_, is the least favorable step, then the minimum overpotential of the HER would be defined as [Eq. (5)]:

(5) $$\eta_{T}=E_{H_{ad}/H_{2}}^{o}-E_{H^{+}/H_{2}}^{o}$$

For the HER and HOR, any catalyst with Δ*G*° (H_ads_)≠0 will have *η*_T_>0. Therefore, the close to thermoneutral chemisorption of hydrogen on Pt (Δ*G*_Hads_≈0 eV) favours its facile interconversion between the H^+^ and H_2_ species at the interface and is responsible for the high exchange current density of HER/HOR on Pt of the order 10^−2^–10^2^ A cm^−2^,^23^ which is at least six orders of magnitude higher compared to that of the O_2_/H_2_O couple on the same metal.

Extending this perspective to more complex reactions involving the transfer of more than two electrons and at least two reaction intermediates, it is expected that any reaction intermediate will have a finite energy of adsorption Δ*G* with its equilibrium potential defined by the Nernst equation. All intermediates with Δ*G*≠0 contribute to the overpotential, although the intermediate with the highest thermodynamic equilibrium potential is expected to contribute the most to *η*_T_. Multielectron transfer reactions with more than one intermediate, therefore inherently exhibit high overpotentials because of the thermodynamic voltage loss (*η*_T_) needed to activate energetic intermediates.^22^, ^24^

#### 2.2.2 Evaluation of Electrocatalytic Activity

**Figure 1:**
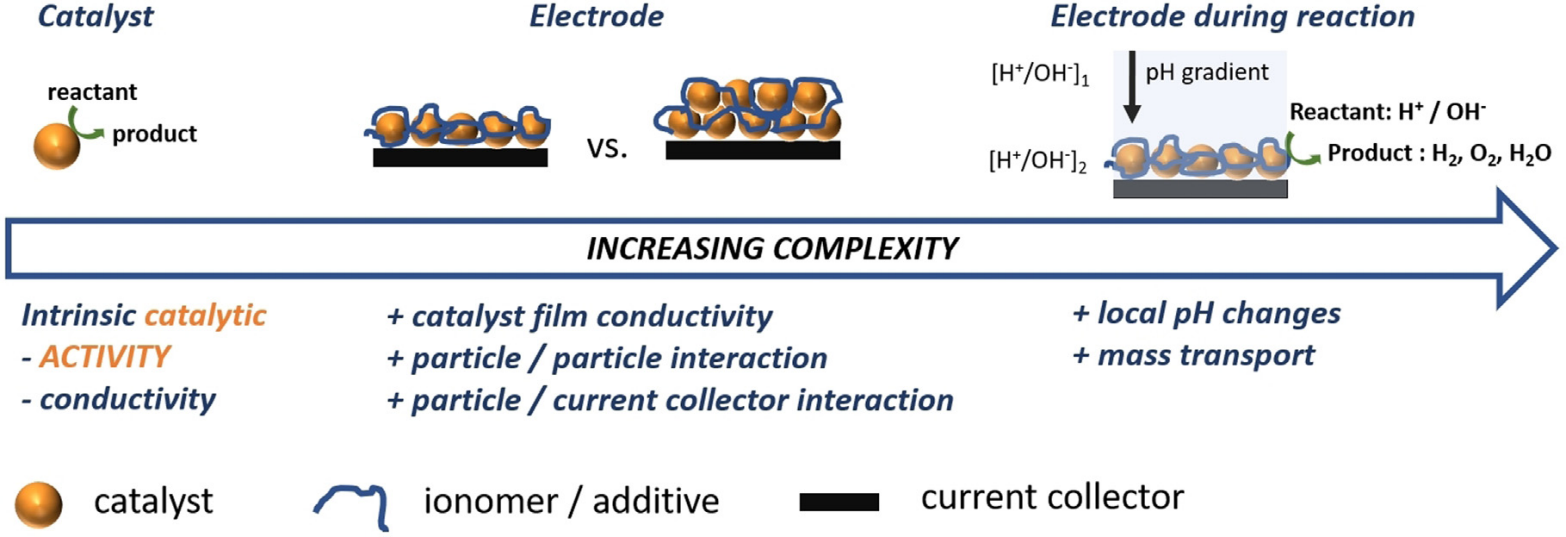
Schematic depiction of a single‐catalyst nanoparticle, a monolayer, and ensemble of catalyst particles immobilized on an electrode surface, for example, by using a binder or ionomer, and a layer of catalyst particles in the presence of an electrolyte during electrochemical conversion.

Most of the materials studied in electrocatalysis come in the form of powders with some having complex compositions. The typical approach for assessing the electrocatalytic activity of such catalyst materials is to disperse them in a suitable solvent containing an ionomer and drop coat a portion of the suspension on an electrode to form a catalyst film similar to what is depicted in Figure 1. The scheme shows catalyst particles as a single entity, as a monolayer and multilayer adsorbed onto an electrode together with an ionomer as binder, and a reaction taking place on an electrode immersed in an electrolyte. Since electrocatalytic currents are typically normalized with respect to the geometric area of the electrode, a desirable situation would be the formation of a monolayer coverage in which the area covered by the catalyst is identical to that of the electrode. For an electrode modified with 2D catalytic films, an electrocatalytic cycle is comprised of mass transport of the reactant and associated ions (anions or cations) from the bulk to the catalyst/electrolyte interface, adsorption of the reactant on the electrode surface, electron transfer between the catalyst and the reactant, and finally, transfer of electrons between the catalyst and the support electrode. Electron transfer between the catalyst film and the support electrode should be fast, which is a major criterion for selection of the electrode. Most films are however formed as multilayers segregated from each other with domains not covered with the catalyst. These catalyst particle films can have a significant thickness and may additionally contain binder and/or conducting additives as well as micro‐ and macropores for increased surface area and facilitating mass transport. This has implications on the reliability of determining the intrinsic activity of the catalyst particles. For such films, not all active catalyst particles will be in contact with the electrolyte or electrically connected, thereby precluding them from participating in the reaction.

The ionomer, not being electronically conductive, may also insulate some of the particles from taking part in the reaction. Upon immersion of the modified electrode in the electrolyte and depending on the properties of the electrolyte and the ionomer, spontaneous interactions may take place and alter the properties of the catalyst. For example, in the case of Nafion, the sulfonate anions are likely to interact with the electrolyte, introducing interactions that are seldom accounted for. These factors, together with the complex phenomena triggered by application of an electric field are expected to interfere with the electrocatalytic reactions. This is even more complex in proton‐coupled electron‐transfer reactions which cause local pH changes leading to different potentials and hence kinetics at different active sites within the catalyst film. Such interactions make it extremely difficult to reliably extract accurate kinetic data concerning the intrinsic activity of the catalyst particles. The impact of complex film properties on the accuracy of determining intrinsic kinetic parameters (*i*_o_, *k* ^o^ and TOF), are for example revealed by stark differences in experimental *i*_o_ values for HER/HOR on Pt determined by RDE voltammetry that are usually at least one order of magnitude less than those determined by ultramicro(nano)‐electrode and scanning electrochemical microscopy (SECM) based techniques.^25^ The kinetics of the HER/HOR on RDE are very fast while mass transport is considerably slower. In contrast, mass transport at ultramicro‐ and nano‐electrodes are significantly faster than in RDEs,^26^ which enables more accurate determination of mass transport free kinetic currents, avoids local pH changes as well as accumulation of reaction products, and consequently is the basis of more accurate values of *i*_o_, *k*_o_, and TOF.

In light of the complications of using large particle ensembles and complex films to determine fundamental electrocatalytic parameters of individual active sites, it is necessary to develop more accurate and reliable alternative methods. In Section 2.2.3, single‐entity electrochemistry and nanoelectrochemistry techniques for more accurate determination of fundamental electrocatalytic parameters are discussed with examples from recent literature.

#### 2.2.3 Single‐Entity and Nanoelectrochemistry

Definitive understanding of the composition, properties, and electrocatalytic activity of individual active sites, or single entities such as nanoparticles, atoms, and molecules, as well as their dynamic behavior under reaction conditions is one of the fundamental goals of electrocatalysis. Nanoelectrochemistry is concerned with probing electrochemical phenomena of and at nanosized domains.^27^ Nanoelectrochemical methods make it possible to isolate and study electrochemical properties of individual nanoparticles and therefore has advantages compared to methods from which inferences about nanoparticle behaviour are drawn from studies of aggregated nanoparticle ensembles. The possibility to fabricate nanosized electrodes with precise control of their diameters^28^, ^29^ and to perform electrochemical investigations either at such electrodes, or after their modification with individual nanoparticles, makes it possible to observe phenomena at the nanoscale free from interferents that compromise the reliability of the data from catalyst film modified macroelectrodes.^28^, ^30^, ^31^

##### 2.2.3.1 Nanoparticle at a Nanoelectrode Tip

A specifically unique feature of electrochemical measurements at nanoelectrodes is the extremely fast mass transport with the RDE equivalent of about 25000 rpm for approximately 5 μm particles, 10^6^ rpm for 1 μm particles, and 10^8^ rpm for ≤50 nm particles.^26^ This feature makes it possible to study kinetic phenomena more reliably without mass‐transport hindrances and local concentration and pH changes. For example, investigation of the ORR on single Pt nanoparticles of different sizes, supported on carbon nanoelectrodes, revealed a particle size‐dependent ORR mechanism. That is, H_2_O_2_ formation was observed to increase with a decrease of the Pt nanoparticle size, which is inconsistent with studies of Pt nanoparticles ensembles on macroelectrodes. Higher H_2_O_2_ formation on the smaller Pt nanoparticles was attributed to faster diffusion of H_2_O_2_ from the nanoparticles before it can be further reduced to H_2_O.^26^

In a study of the OER on different nanosized Ni(OH)_2_ particles in the size range from 20 to 500 nm electrodeposited on carbon nanoelectrodes, the TOF was observed to decrease with increase in Ni(OH)_2_ particle size. The obtained TOFs were one to three orders of magnitude higher than reported for experiments performed on macroelectrodes.^30^ Besides faster mass transport, three additional factors render the TOFs of the OER on such nanoparticles to be more reliable compared to those reported from macroelectrode measurements. Firstly, the contribution of charging effects on the measured current is negligible. Secondly, since neither binder nor conductive additives are used, the entire nanoparticle is expected to be electronically well connected. Lastly, the actual number of active sites used for determination of TOF is expected to be more accurate for such a single nanoparticle since their sizes can be determined more accurately.

**Figure 2:**
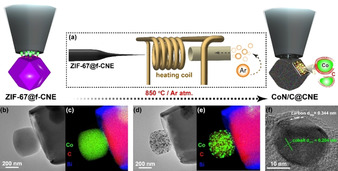
a) Setup used for pyrolysis of the ZIF‐67@f‐CNE nano‐assembly. b) TEM image of the ZIF‐67@f‐CNE nanoassembly and c) corresponding EDX elemental intensity maps. d) TEM image of the resulting CoN/C@CNE nanoassembly and e) corresponding EDX elemental intensity maps. f) Representative TEM image of a Co–C (core–shell) nanoparticle embedded inside a CoN/C matrix. Image taken from Ref. 31 with permission of the publisher, Wiley‐VCH.

Metal–organic frameworks (MOFs) are of topical interest in electrocatalysis. However, because of their lack of electronic conductivity, MOFs by themselves are typically not suitable for electrocatalytsis. When pyrolyzed, MOFs decompose to form metallic or metal oxide nanoparticles partially embedded in a N‐doped graphitic carbon matrix.^31^ Figure 2 shows an example of a MOF particle grown at the tip of a carbon nanoelectrode. The MOF nanoparticle (Figure 2 b,c) was pyrolyzed in a custom‐built setup (Figure 2 a) to form a Co–C (core–shell) nanoparticle embedded inside a CoN/C matrix. When evaluated for the OER in 0.1 m KOH, a high current density of 230 mA cm^−2^ at 1.77 V (vs. RHE) and a remarkably high turnover frequency of 29.7 s^−1^ at 540 mV overpotential were reported, both rarely observed on macroelectrodes. Post‐electrocatalysis TEM analysis of the nanoparticle coupled with EDX elemental mapping (Figure 2 b–e) disclosed coalescence of the Co sites into larger agglomerates thus unravelling insights into the dynamic changes of individual nanoparticles during OER, which is rarely accessible from macroelectrode measurements.^30^

##### 2.2.3.2 Nanoimpact Electrochemistry

Nanoimpact electrochemistry involves the immersion of a stationary electrode under potential control into an electrolyte solution containing freely diffusing nanoparticles and studying the electrochemical phenomena that occur during stochastic impact of individual nanoparticles with the electrode.^32^ Nanoimpact electrochemistry makes it possible to determine the size of individual nanoparticles impacting an electrode and to obtain kinetic data during a catalytic event, which does not only greatly simplify studies of the dependence of electrocatalytic activity on nanoparticle size but also yields more accurate data as opposed to inferences drawn from ensemble studies. Most of the earlier studies of electrocatalytic events during nanoparticle‐electrode impacts were mostly qualitative or limited to determination of nanoparticle sizes,^33^, ^34^ since accurate extraction of kinetic data has to account for complex dynamic phenomena that occur prior to, during, and after the impact, that are not necessarily trivial to address.^35^ In a study of proton reduction by 10 nm gold nanoparticles by nanoimpact electrochemistry, the recorded currents were observed to be highly variable, which was attributed to nanoscopic motion of the nanoparticles during their contact with the electrode leading to differing connectivity of the nanoparticles to the electrode.^36^ Proper electronic contact, which is in turn dependent on the residence time of the nanoparticle has been identified as a limiting factor for determination of accurate kinetic data.^34^, ^37^ Nonetheless, initial attempts at using this technique to determine kinetic parameters are very promising. A recent kinetic study of the intrinsic OER activity of 4 nm sized CoFe_2_O_4_ spinel nanoparticles by nanoimpact electrochemistry disclosed an unprecedentedly high TOF of 2×10^5^ s^−1^.^38^

The ability of nanoimpact electrochemistry to simultaneously determine nanoparticle size and kinetic data of reactions catalyzed by single nanoparticles presents a unique tool for elucidating the dependency of electrocatalytic performance on nanoparticle size, morphology, and structure. Poor electronic contact and particle agglomeration are some of the main drawbacks, together with the limited bandwidth of low‐current potentiostats that must be overcome to make this technique more versatile in studying electrocatalysis at single nanoparticles.

##### 2.2.3.3 Scanning Electrochemical Cell Microscopy (SECCM)

SECCM is a scanning probe technique with integrated features of an electrochemical droplet cell, where electrochemical measurements are performed in a microdroplet of electrolyte formed between the tip of a microcapillary and the surface to be investigated.^39^ An exceptional capability of SECCM is its ability to study surface structure–activity dependencies at the nanoscale. By combining the power of nanoelectrochemistry and scanning microscopy, SECCM is well‐suited to facilitate direct visualization of spatially resolved reactivity of surfaces arising from compositional differences and morphological variations, including, grain boundary effects, steps, terraces, and edges, among others, which are impossible to observe using macroscopic techniques.^40^, ^41^ For example, it was revealed by SECCM that the edge planes of MoS_2_ are more active than the basal planes in catalyzing the HER.^42^ Using SECCM, Mariano et al. successfully observed that the grain boundary surface terminations in gold electrodes were more active for electrochemical CO_2_ reduction to CO compared to grain surfaces.^43^ In the scanning mode, SECCM can be used to map the electrochemical behavior of individual nanoparticles in a nanoparticle ensemble thereby enabling subtle peculiarities other than nanoparticle size such as orientation, exposed facets,^41^ effect of particle agglomeration, and support–nanoparticle interactions, among others, to be resolved.^44^, ^45^

The exchange current density of the HER determined using SECCM on the basal plane of MoS_2_ was 2.5×10^−6^ A cm^−2^, whereas it was 40 times higher on the edge plane of MoS_2_ (1×10^−4^ A cm^−2^).^46^ Small variations in the surface structure and composition of a single Fe_4.5_Ni_4.5_S_8_ nanoparticle resulted in a change in the HER activity that could be discerned by SECCM.^47^ In a study of the HER on holey graphene (G) by SECCM, the edges were identified to be the most active sites. The HER increased upon doping G with N or P and was highest when G was co‐doped with both N and P (NP). Moreover, the HER was faster on NP co‐doped G with edge structures, with a TOF of 0.64 H_2_ s^−1^ at −200 mV (vs. RHE) compared to its edge‐free counterpart with a TOF of 0.45 H_2_ s^−1^.^48^ Most of the studies were performed in acidic electrolytes because of the favorable wetting properties. Recently, by using a soluble reversible redox species, a N_6_‐coordinated Os complex, as an internal standard together with a rigorous measuring regime, TOF values in the range of 0.25 to 1.5 s^−1^ per Co atom in the potential range from 1.70 to 1.80 V (versus RHE) were determined for individual ZIF‐67 MOF derived Co‐C particles for the OER.^45^ SECCM has therefore firmly asserted itself as a robust tool for direct imaging and quantitative study of the reactivity of surfaces with nanoscale capability to resolve composition and morphology dependent electrocatalytic activity of surfaces and individual nanoparticles.

### 2.3 Stability

Undervaluation of catalyst stability is, to a large extent, responsible for the wide gap between apparently exciting breakthroughs in designing active catalysts and the practical implementation of such catalysts in functional devices. This discordance is mainly due to lack of well‐defined guidelines and benchmarks for rigorous assessment of catalyst stability in view of potential applications. It is vital that catalyst stability and activity are considered integrally to determine mutual activity–stability determinant properties and factors that can be concurrently optimized to accelerate the translation of important breakthroughs in material design into application. Consideration of the thermodynamic and kinetic stability of materials under the conditions of their envisaged application is a key requirement for designing stable catalysts. Pourbaix diagrams showing the potential–pH dependence of chemical species are therefore a good initial source of reference. However, it is important to consider the aforementioned local pH value modulations during electrocatalysis, especially at high turnover at high current densities.

There are recent interesting efforts aimed at establishing unified activity–stability relationships that can simplify the identification of materials with the right activity–stability balance. For the OER, Kim et al.^49^ proposed a quantitative parameter [Eq. (6)] that expresses the activity–stability dependence of catalysts called the activity stability factor (ASF), where *I* is the rate of oxygen generation given by the current density at a specific overpotential (*η*) and *S* is the rate of dissolution of the catalyst given by the dissolution current density.

(6) $$ASF=\;{\left.\frac{I-S}{S}\right|}_{\eta }$$

Since the goal is to achieve a high rate of oxygen generation with minimum dissolution of the metal, the higher the ASF the better the activity–stability performance of a catalyst. Here, determination of the ASF requires simultaneous measurement of *I* and *S*, which was achieved by coupling a stationary probe rotating disk electrode (SPRDE) to an online inductively coupled mass spectrometer (ICP‐MS).^49^ A similar activity–stability metric, called the stability number (S‐number), essentially similar to ASF but expressed somewhat differently in gravimetric terms as the ratio of the amount of evolved oxygen to the amount of dissolved catalyst was reported by Geiger et al.^50^ Although a scanning flow cell (SFC) coupled to online ICP‐MS detection was used in the latter method, and without disregard to its high sensitivity resulting from measurements in minute electrolyte volumes, its main advantage is that the amount of evolved oxygen and dissolved metal do not necessarily have to be measured simultaneously and the dissolved metal analyser does not have to be necessarily coupled to an electrochemical flow cell system. This feature appears to make it possible to use relatively simple and easily available techniques, such as the rotating‐ring disc electrode (RRDE), to determine activity–stability correlations.^51^

#### 2.3.1 Catalyst Sability During Electrochemical Reduction

Most metals are predicted to be thermodynamically stable in aqueous electrolytes at low electrode potentials as used for the HER, CO_2_RR, and NRR. Under these conditions, instability is expected to be mainly driven by physical phenomena, including vigorous gas evolution leading to physical detachment of catalyst particles, especially in the case of nanoparticle‐based catalysts. However, Ru and Ir were both reported to dissolve in alkaline and acidic electrolytes under the HER conditions, coinciding with the reduction of their native oxides.^52^ Since the Pourbaix diagrams of Ir and Ru indicate that under these conditions both Ru^0^ and Ir^0^ are expected to be thermodynamically stable species, it is also possible that the dissolution could have been instigated by physical phenomena as a result of the evolved gas pressure and convective forces of electrolyte flow in the flow system. Related physical factors could also account for the reported dissolution of Cu during electrochemical CO_2_ reduction, at conditions under which Cu^0^ would otherwise be expected to be thermodynamically stable.^53^ In contrast, the ORR half‐reaction proceeds at potentials at which most metals, and also carbon as a common support, are predicted to be thermodynamically unstable. In alkaline electrolytes, most transition metals as well as the less noble metals (Ag and Au), tend to undergo surface oxidation to form a self‐passivating hydroxide layer that increases their kinetic stability, thus significantly slowing their dissolution. By contrast, most metals, except the platinum group metals (Pt, Pd Ir, Rh and Os), exhibit both low thermodynamic and kinetic stability in acidic electrolytes and can therefore not serve as ORR catalysts in low‐pH electrolytes.

#### 2.3.2 Catalyst Stability during Electrochemical Oxidation

As the HOR and OER half‐cell oxidation reactions are of topical importance in energy conversion cycles, (electro)chemical stability is discussed in relation to these two reactions, but the underlying phenomena should be applicable to the oxidation of other molecules, such as alcohols under similar conditions, albeit taking into consideration their unique reaction mechanisms and intermediates. The HOR is fast on Pt group metals and its reaction kinetics decrease with electrolyte pH. Most metals suffer rapid dissolution in acidic electrolytes. The decrease of the HOR kinetics with electrolyte pH is associated with the role of dissociated interfacial water or M‐OH_ad_ adsorption.

**Figure 3:**
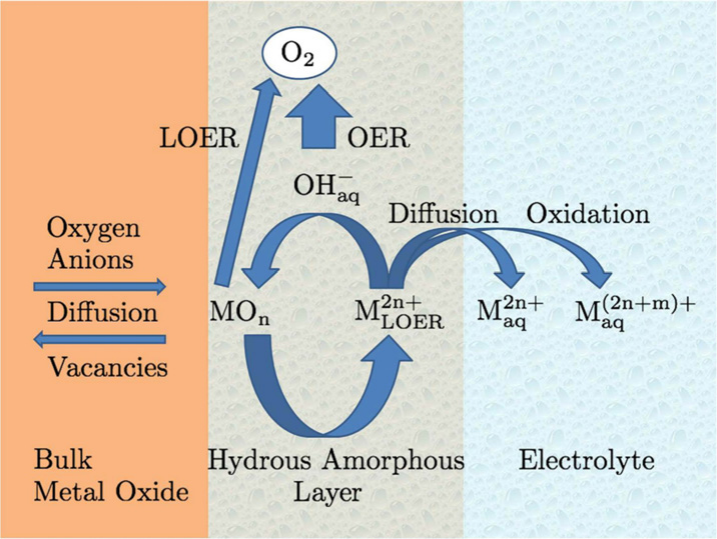
Schematic representation of the proposed lattice oxygen evolution reaction (LOER) cation cycle. The $M_{LOER}^{2n+}$ cation can either react with OH^−^ anions from the electrolyte to evolve oxygen or dissolve in the electrolyte with unchanged oxidation state or after oxidation to a higher oxidation state. Taken from Ref. 54 with permission of Springer Nature.

All surface metal atoms or ions of metals and metal oxides are oxidized to a higher oxidation state (e.g. ≥3+ for Co and Ni, and ≥5+ for Ru and Ir) of inherently lower thermodynamic stability prior to the OER. It has been shown by online electrochemistry/ICP‐MS analysis and by RRDE^49^ that more pronounced dissolution coincides with the transition from a lower to a higher oxidation state (M^*m*+^→M^*n*+^) prior to O_2_ evolution although dissolution also generally occurs at lower potentials than required for M^*m*+^→M^*n*+^ transition. Binninger et al. have shown on the basis of thermodynamic considerations that the thermodynamic driving force or overpotential required for disintegration of metal oxides to release lattice oxygen (*η*_LOER_) with likely concomitant dissolution of the metal (Figure 3) is greater than necessary for the OER (*η*_OER_), from which they concluded that all metals are in a state of thermodynamic instability during the OER.^54^

The detection of lattice oxygen in the evolved oxygen gas during the OER by means of isotope labelling therefore serves not only as a prognosis of the LOER mechanism but also as a marker for the instability of metal oxide catalysts. The mechanism of O_2_ evolution by the LOER mechanism is essentially similar to the conventional OER mechanism except that the oxidation state of the metal active sites (MO_*n*_) does not change prior to and during O_2_ evolution.

**Figure 4:**
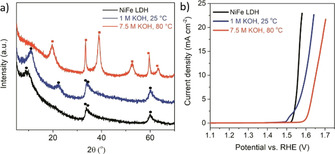
a) X‐ray diffractograms registered for NiFe‐LDH (black) and NiFe‐LDH after immersion for 60 h in 1.0 m KOH at 25 °C (blue) and 7.5 m KOH at 80 °C (red) (• NiFe CO_3_^2−^, ▪ β‐Ni(OH)_2_). b) Linear sweep voltammograms for NiFe‐LDH (black) and NiFe‐LDH after immersion for 60 h in 1.0 m KOH at 25 °C (blue) and 7.5 m KOH at 80 °C (red) registered at a scan rate of 5 mV s^−1^ and 1600 rpm. Taken from Ref. [DOI: https://doi.org/10.1002/chem.201803165] with permission of the publisher, Wiley‐VCH.

From the perspective of potential industrial applications, rigorous test criteria for material stability and durability at, or as close as possible to, industrial‐relevant conditions should replace the few minutes to a few hours tests that predominate in the current reports. For example, an investigation of the chemical stability of NiFe double layer hydroxide (NiFe‐LDH), one of the most active electrocatalysts for the OER in alkaline electrolytes, by its immersion in 1 m and 7.5 m KOH at 80 °C for 60 hours revealed profound structural transformation of the material accompanied by a drastic decline of the OER activity, even in the absence of any applied electrochemical potential (Figure 4).

XRD analysis disclosed a decrease in the basal spacing of the NiFe‐LDH structure, after treatment in 1.0 m KOH (80° for 60 h), as indicated by a shift of the (003) reflection to a higher value (2*θ*=11.2°), which was ascribed to the replacement of the NO_3_^−^ anions with either CO_3_^2−^ or OH^−^ ions. The sample treated in 7.5 m KOH (80° for 60 h) underwent complete chemical and structural transformation into a mixture of crystalline β‐Ni(OH)_2_, as detected by XRD, and amorphous FeOOH and minor crystalline domains of α‐FeOOH that were revealed by HRTEM and SAED. As shown in Figure 4 b, there was drastic decline in OER activity accompanying these structural changes. The overpotential required to attain a current density of 10 mA cm^−2^ increased from 340 mV for pristine NiFe‐LDH to 370 mV and 420 mV for the NiFe‐LDH samples treated in 1.0 m KOH and 7.5 m KOH, respectively. Furthermore, the Tafel slope of the OER changed markedly, increasing from 52 mV dec^−1^ for the pristine sample to 118 mV dec^−1^ and 148 mV dec^−1^, respectively, for the samples treated in 1.0 m KOH and 7.5 m KOH at 80 °C for 60 h, indicating significant changes in the reaction mechanism.^81^

In a related study, the chemical stability of nickel phosphide (Ni_*y*_P) particles of different sizes was investigated by immersing the catalyst particles in 1.0 m KOH at 80 °C for different durations up to 168 hours. Under these purely chemical conditions, without applying any polarization, phosphorus was observed to continually dissolve from the catalyst particles, leading to formation of Ni_*y*_P@NiO_*x*_H heterostructures accompanied by a loss in activity of the OER.^82^

These examples demonstrate that the composition and properties of catalysts at the conditions of their envisaged application, that is, high electrolyte concentrations and temperature, can change drastically, and in a detrimental way, from those at room temperature. Therefore, a few minutes or hours of stability tests at room temperature are not representative of their possible behavior under industrial conditions. Meaningful demonstration of material stability and durability should therefore mimic the realistic conditions of the envisaged application. Moreover, we propose that each newly discovered catalyst material should be subjected to an initial stability test by immersing it into the electrolyte at the temperature of the envisioned application before performing optimization of activity for a given reaction.

### 2.4 Selectivity

For reactions with multiple possible products, the ability to control selectivity to obtain a specific product(s) presents one of the greatest challenges. Many factors influence product selectivity, including applied electrode potential or current, properties of the catalyst, and the electrolyte pH, among others. Reactions often involve multiple possible reaction pathways and intermediates, so a crucial prerequisite toward gaining insightful control over the product selectivity is a solid understanding of all the possible reaction pathways and the underlying reaction mechanisms, and how they are influenced by various reaction parameters.

**Figure 5:**
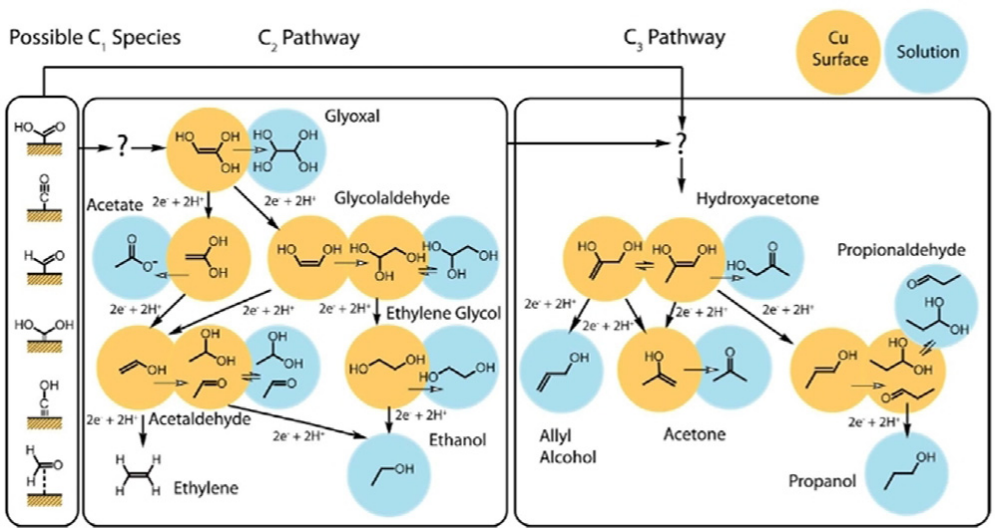
Proposed reaction pathways of the CO_2_RR for C_2_ and C_3_ products with enol‐like surface intermediates. Arrows between overlapping circles indicate changes between the enol, keto, and diol form of each product. Arrows between non‐overlapping circles indicate electrochemical reduction steps involving the addition of 2 H^+^ and 2 e^−^. For simplification, product names are intended to refer to all forms of the product. Taken from Ref. 55 with permission of the Royal Society of Chemistry.

As an example, for electrochemical CO_2_ reduction, where up to 16 different products are possible,^55^ the mode of adsorption and activation of the CO_2_ molecule at the initial stage of the reaction is reported to be the most decisive factor. The most economically valuable products are those rich in carbon (C≥2). Many of these products however tend to be formed at potentials where the HER is also active. Thus, H_2_ formation is a notorious side‐reaction that has to be suppressed to favor the formation of carbon‐rich (C_≥2_) products. The formation of C_2_ and higher products involves a reaction pathway that favors C−C coupling, which is highly dependent on the properties of the catalyst (Figure 5).

Copper remains the most investigated catalyst for electrochemical CO_2_ reduction because of its ability to promote C−C coupling mechanisms under formation of multi‐carbon products, for example, ethylene and ethanol.^56^ Previous research has therefore mostly focused on tuning the properties of Cu through strategies such as alloying and morphology controlled nanostructuring. The drawback with this approach, and all other approaches that primarily focus on first identifying catalysts exhibiting a high faradaic current and then attempting to tailor the selectivity afterwards, is that catalysts that exhibit low faradaic currents, and may otherwise be intrinsically more selective towards specific products, are likely to be missed.

New catalyst classes that promote the formation of C_2_ and higher products, including C_3_ and C_4_, are beginning to emerge. Calvinho et al. reported the formation of C_3_ and C_4_ oxyhydrocarbons, including methylglyoxal (C_3_) and 2,3‐furandiol (C_4_), at relatively low overpotentials when nickel phosphide (Ni_2_P) was employed as the catalyst.^57^ FeP supported on a titanium mesh was reported to selectively reduce CO_2_ to ethanol (FE≈80 %) and methanol.^58^ Boron‐doped Cu selectively reduced CO_2_ to C_2_ products with a faradaic efficiency of about 79 %, with boron believed to stabilize the ratio of Cu^δ+^/Cu^0^ surface species, thereby enhancing selectivity towards C_2_ products.^59^ A Au/Cu catalyst composed of Au nanoparticles on Cu foil, exhibited selective conversion of CO_2_ into C_2_ alcohols (ethanol and *n*‐propanol). In this tandem catalyst, CO_2_ is first converted into CO close to Cu sites that subsequently convert CO into C_2_ alcohols.^60^ A Ni‐Ga catalyst reduced CO_2_ to highly reduced products, including methane, ethylene, and ethane, at low overpotentials.^61^

A detailed understanding of the properties of such materials and the mechanisms through which they promote the formation of such products is expected to lead to exciting new discoveries. A paradigm change where high selectivity towards a desirable product takes precedence over faradaic activity may offer certain advantages, such as low product separation costs. The fact that even for a single catalyst material, multiple factors including particle size and morphology, degree of crystallinity (single crystal or polycrystalline), exposed facets, etc.,^62^ also influence product selectivity makes insightful prediction or product selectivity very challenging, even for computational modelling.

Surmising from the enormous effort dedicated towards the development of efficient catalysts for the most commonly investigated electrochemical reactions, and the slow pace of progress, it becomes apparent that further progress and a significant breakthrough on a scale necessary for commercial deployment of developed catalysts is unlikely to be realized through optimization efforts that have been ongoing for the last 20 years. We argue that only a paradigm shift in catalyst design can cause the step‐change necessary for the next generation of catalysts. In the following, we discuss some promising emerging catalyst design concepts that are worth highlighting.

Surfaces with identical active sites do not seem to be suited for electrocatalysis of multistep reactions with a wide range of possible products, wherein the adsorption energies of the reaction intermediates have a wide distribution. In cascaded electrocatalysis, the active sites of an electrocatalyst are spatially arranged in a such a way that an intermediate or product generated during a specific electrochemical step at one of the active sites is subsequently converted at the adjacent active site through either surface diffusion, forced convection, or confinement. In flow electrochemical reactors, active sites are spatially separated so that a product formed upstream is transported to downstream active sites by convection.^63^ We give a brief overview on high‐entropy alloys (HEAs), nanozymes, and nanocavities by design, as emergent catalyst design concepts that facilitate cascaded electrocatalysis to achieve unprecedented selectivity of desired products.

#### 2.4.1 High‐Entropy Alloys (HEAs)

High‐entropy alloys (HEAs) are alloys formed by the combination of at least five metals. The properties of HEAs vary considerably depending on the alloy composition, for example, from being paramagnetic to superparamagmetic, and some HEA exhibit superconductivity. Interestingly, it has recently been discovered that some HEAs exhibit very intriguing electrocatalytic properties.^64^, ^65^ Owing to their complex composition and the atomic scale proximity of various atoms with a continuous distribution of adsorption energies, HEAs might finally be the solution to overcoming the conundrum of scaling relationships. For the ORR, the key reaction intermediates, OOH and OH, exhibit similar adsorption energies on a given surface since they both bind to the surface via the oxygen atom. Therefore, any attempt to selectively optimize the adsorption energy of one of these species without affecting the other in a similar way is impossible, which is the problem of the scaling relationships.^66^ DFT calculations by Rossmeisl's group on a HEA comprising IrPdPtRhRu, showed that in contrast to single active sites that display discrete adsorption energies for O* and OH* intermediates, the HEA exhibits a nearly continuous distribution of adsorption energies.^64^ A multinary alloy comprising CrMnFeCoNi, prepared by co‐sputtering of the target elements into an ionic liquid, was reported to exhibit a high intrinsic ORR activity in 0.1 m KOH similar to that of Pt. The removal of any one of the elements from the alloy resulted in a significant drop of activity, thereby underscoring a cooperative electrocatalytic effect necessitating interdependent interaction of all the five elements.^67^

**Figure 6:**
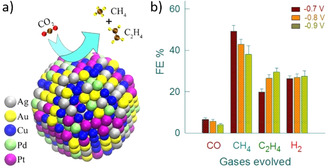
a) Schematic representation of the HEA alloy composition and the conversion of CO_2_ into CH_4_ and C_2_H_4_. b) A bar diagram showing the faradaic efficiencies of CO, CH_4_, C_2_H_4_, and H_2_. Taken from Ref. 71 with permission of the American Chemical Society.

HEAs have also been reported to efficiently catalyze the OER,^68^ the HER,^69^ as well as electrochemical reduction of CO and CO_2_.^70^, ^71^ For electrochemical CO_2_ reduction, the prospect of tuning the surface reactivity of HEAs by changing their composition makes it possible to tailor the surface sites optimized for specific products. A HEA comprising AuAgPtPdCu was reported to achieve CO_2_ conversion into gaseous hydrocarbons, including CH_4_ and C_2_H_4_, with a high faradaic efficiency close to 100 % (Figure 6), and it was attributed to a reversal in the adsorption trends of two intermediates, *OCH_3_ and *O, out of eight possible intermediates, compared to the their adsorption on a Cu(111) surface.^71^

The emergence of HEA seems to fulfil the concept of a designer surface envisioned by Bockris and Minevski, where different patches of a multielement surface are optimized to catalyze specific steps in a complex reaction mechanism involving many intermediates.^72^ With each atom surrounded by at least four other dissimilar atoms in its close vicinity, the unique electrocatalytic properties of HEAs are attributed to the unique interactions of the neighburing elements, and their ability to act concertedly in an interdependent manner. Moreover, the ability to tune their properties by varying the alloy composition means that tailored surfaces seem to make it possible to tune not only the kinetics of a reaction, but also its selectivity and stability, particularly for complex multistep reactions. However, in‐depth understanding of how the nature of the interactions among the elements in a given HEA impact their surface properties and ultimately the mechanism of a given reaction are essential to make further advances. Such an ambitious goal seems to be within reach considering the fast pace of progress in catalyst design by deploying robust computational algorithms and deep machine learning.^73^

#### 2.4.2 Electrocatalysis by Nanozymes and in Nanoconfined Volumes

Enzymes achieve highly selective conversion of their substrate at active sites that are often buried inside the protein matrix, with channels containing charge‐transfer centers leading to the active sites. The high selectivity of an enzyme is due to their ability to confine the substrate and reaction intermediates within the enzyme channel and thereby facilitate its stepwise cascaded catalysis into the final product.

A nanozyme is a nanoparticle designed to mimic the functionality and 3D structure of enzymes.^74^ Although the concept of nanozymes has been applied in other fields of research, for example in sensing, for some years, deliberate design of such particles for electrocatalytic applications is relatively new. In a recent study, nanozymes were fabricated by selectively etching Ni from Pt‐Ni nanoparticles to create isolated substrate channels in the nanoparticles, where the channels do not interact with each other. When investigated for the ORR in 0.1 m HClO_4_, the nanozymes exhibited superior activity with a turnover frequency that was three times higher than that of a benchmark Pt‐Ni catalyst.^75^

**Figure 7:**
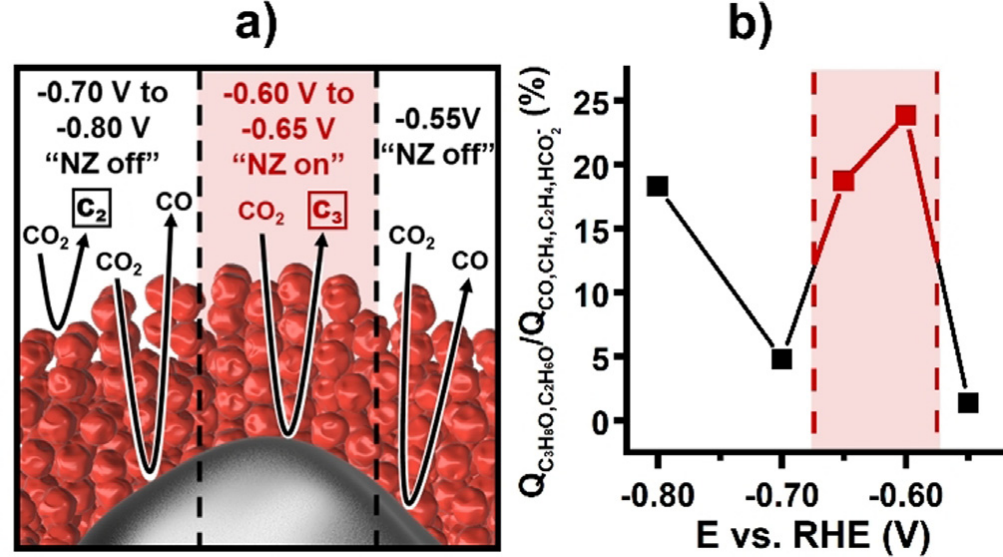
a) Schematic representation of processes occurring at specific potential windows with active (red) or inactive (black) nanozyme cascade mechanism. b) Preference for the formation of C_2_ and C_3_ products over other carbon‐containing products as charge ratio. Nanozyme activity window is marked in red. Taken from Ref. 76 with permission of the American Chemical Society.

Nanozymes can be designed to tailor reaction selectivity and enhanced product turnover by having active sites that favor specific reaction steps or conversion of a targeted intermediate spatially arranged to facilitate cascaded reactions through sequential reactions or deliberate nanovolume confinement. This concept was applied to design nanozymes comprising a Ag core and a porous Cu shell, where CO_2_ is converted at the Ag core into CO, while the copper pores not only reduce CO further but also locally increase the concentrations of CO_2_ and CO to achieve enhanced overall turnover (Figure 7). Significantly enhanced selective reduction of CO_2_ towards C_≥2_ products was achieved in this proof of concept study.^76^

Catalysts with nano‐ or microcavities designed to constrain mass transport and thus increase the duration of interaction of substrates with the active sites can potentially enhance substrate conversion as well as selectivity.^77^ Since several extrinsic factors, including nanoparticle size, pore diameter, and electrolyte pH, among others, are known to also influence the turnover rates and reaction selectivity, there is plenty of room for optimization of cascaded electrocatalysis using nanozymes and electrocatalysis in nanoconfined volumes to progressively improve turnover and tailor the selectivity of multiproduct reactions.

**Figure 8:**
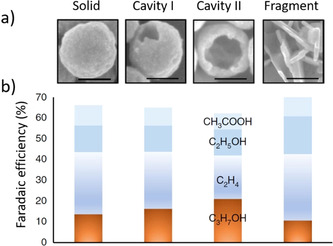
Representative SEM images of a solid copper nanoparticle, two copper nanocavities of different sizes, and copper fragments. The scale bars are 100 nm. Faradaic efficiencies of C_2_ and C_3_ products for the corresponding particles shown in the top frame. Take from Ref. 78 with permission of Springer Nature.

In a particular example involving the electrochemical reduction of CO using copper nanocavities as the catalyst, the faradaic efficiency for C_3_H_7_OH formation was higher for the catalyst particles with cavities compared to those without, and it increased with the size of the cavities (Figure 8).^78^ The formation of C_3_ products is believed to have been possible because of the confinement of the C_2_‐reaction intermediates within the activated nanocavities, thereby facilitating C_2_–C_1_ coupling to C_3_ products. In a comparative study of the ORR on flat and nanoporous Au surfaces, higher H_2_O_2_ formation was observed on the flat surfaces while the nanoporous gold surface favored the reduction of O_2_ to OH^−^ because of the confinement of H_2_O_2_, thus allowing its further reduction to OH^−^.^79^

The pores of nanozymes or cavities of such nanoparticles can be further tailored with specific functional groups to tune their reactivity and mass‐transport properties, and ultimately the mechanism and selectivity of a given reaction. In a study of the ORR catalyzed by nanozymes comprising Pt‐Ni nanoparticles covered with a surfactant, where Ni was selectively etched to create nanochannels, the specific activity of the ORR was observed to be higher for narrower channels, which was attributed to a higher concentration of protons confined within the channels.^80^

## 3 Summary and Outlook

Advances in understanding the key processes and mechanisms of reactions of key importance in electrochemical energy conversion, complemented by the availability of more reliable characterization techniques, have enabled unprecedented elucidation of structure–performance descriptors leading to remarkable progress in the rational design of active, stable, and selective electrocatalysts. There are still weaknesses in both electrochemical and material characterization methods that prevent accurate determination of critical kinetic parameters and material properties, and the dynamic physicochemical phenomena under given reaction conditions. Developing experimental and theoretical models that capture the dynamic nature of catalysts under given reaction conditions and the associated tandem processes in the electrochemical double layer would facilitate further advances in the field. Improvements in the sensitivity, response time, and data acquisition speed of present‐day operando characterization methods, including near ambient XPS, liquid‐cell electrochemical TEM, electrochemistry coupled with Raman, surface‐enhanced Raman, as well as ATR‐FTIR spectroscopy, and X‐ray absorption spectroscopy, among others, to track not only the steady state, but also transient nature of electrocatalysts and the electrochemical double layer are necessary for in‐depth understanding of reaction mechanisms and design of improved catalysts and electrochemical interfaces. To accelerate the implementation of vital breakthroughs in technological applications, it is important that new experimental perspectives and benchmarks for activity, stability, and selectivity are defined at conditions that realistically depict those expected for the industrial application. As a minimum recommendation, mere immersion of catalysts in concentrated electrolytes at industry relevant temperatures for several hours followed by thorough investigation of composition and structural changes can provide very insightful information on the chemical stability of the catalyst. For fundamental investigations, single‐entity electrochemical methods offer practical and more reliable alternatives to ensemble‐based techniques for evaluation of intrinsic electrocatalytic parameters, for example, TOF, which is necessary for identifying the most potently active sites and hence better knowledge‐guided design of improved catalysts. Single‐entity electrochemical measurements owe their advantage to the extremely fast mass transport and absence of binders and conductive additives that make it possible to determine kinetic currents more reliably.

Presently, preselection of materials exhibiting a high faradaic current prior to investigation and optimization of their selectivity is the dominant approach of searching for selective electrocatalysts. A shift to a design paradigm where emphasis on selectivity takes precedence over the efficiency of electricity‐to‐chemical conversion efficiency may offer striking and viable advantages, including low product separation costs, that might compensate for lower current efficiencies. Besides advances in material design, simultaneous progress in theoretical models, understanding of reaction pathways, and advances in operando characterization tools for identification of reaction intermediates should complement downstream product analysis as a basis for insightful control of reaction selectivity.

## Conflict of interest

The authors declare no conflict of interest.

## Biographical Information

*Justus Masa is a staff scientist at the Max Planck Institute for Chemical Energy Conversion. He received a B.Sc. in Industrial Chemistry in 2003 and a M.Sc. in Chemistry in 2008 from Makerere University. He earned a PhD in Chemistry with Prof. W. Schuhmann at Ruhr University Bochum in 2012. He was a Visiting Scholar at Oxford University in 2013 and a group leader (2015–2019) for Electrocatalysis and Energy Conversion in the Department for Analytical Chemistry and Centre for Electrochemical Sciences (CES), Ruhr University Bochum*.

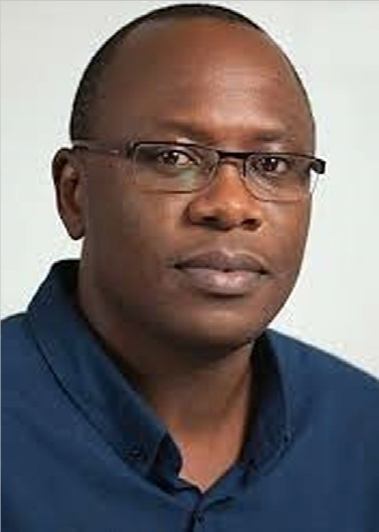

## Biographical Information

*Corina Andronescu received her B. Sc. and M. Sc. from the University Politehnica of Bucharest in 2009 and 2011, respectively, where she also obtained her PhD in 2014 with Prof. H. Iovu. In 2016 she joined the group of Prof. W. Schuhmann (Ruhr University Bochum) first as a postdoctoral researcher and later as a group leader. In 2018, she was appointed Junior Professor at the University of Duisburg‐Essen, where she leads the Electrochemical Catalysis group within the Faculty of Chemistry*.

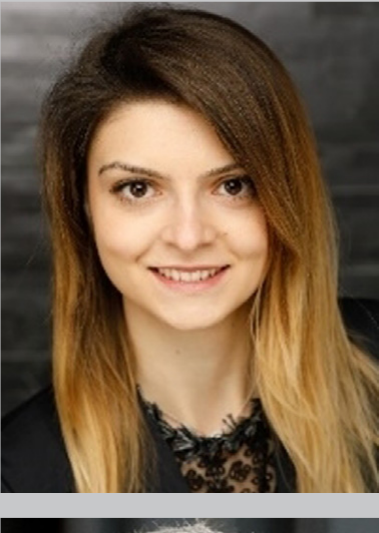

## Biographical Information

*Wolfgang Schuhmann studied chemistry at the University of Karlsruhe, and completed his PhD with F. Korte in 1986 at the Technical University of Munich. After finishing his habilitation at Technical University of Munich in 1993 with Prof. H.‐L. Schmidt, he was appointed Professor of Analytical Chemistry at the Ruhr University Bochum in 1996. His research interests cover a broad spectrum of different fields of electrochemistry*.